# Life in a University Crystallography Service Facility

**DOI:** 10.1063/4.0001098

**Published:** 2025-10-27

**Authors:** Carla S lebodnick

**Affiliations:** Virginia Tech, Blacksburg, VA, USA

## Abstract

I was introduced to crystallography during my PhD research where I studied the structure-function relationship of biomimetic porphyrin complexes at Northwestern. Although crystallography was a tool that I used constantly, I had absolutely no plans to become a crystallographer. I was going to be a chemist! So, for the next stage of my career I completed a postdoc at the University of Michigan working on a project that focused mainly on inorganic synthesis, kinetic studies, and heteronuclear NMR, with absolutely no crystallography. But, life happens - dual careers and job offers from Virginia Tech, where I was hired to establish a crystallography service center in the Chemistry Department. Now, 27 years later, I am still at Virginia Tech and, although my job description is technically the same, much has changed. But, I can say that I proudly call myself a crystallographer. For my short presentation, I will cover factors that I considered when making career decisions and give an overview of my life and job responsibilities as a service crystallographer.

